# Calcitonin Gene–Related Peptide Monoclonal Antibodies and Risk of SARS-CoV-2 Infection and Severe COVID-19 Outcomes Among Veterans With Migraine Disorder

**DOI:** 10.1001/jamanetworkopen.2023.26371

**Published:** 2023-07-31

**Authors:** Kaicheng Wang, Brenda T. Fenton, Yanhong Deng, Sarah E. Anthony, Vinh X. Dao, Emmanuelle Schindler, Richard B. Lipton, Alexander Guirguis, Melissa Skanderson, Elizabeth K. Seng, Jason J. Sico

**Affiliations:** 1 Research, Education, Evaluation and Engagement Activities Center for Headache, Headache Centers of Excellence, US Department of Veterans Affairs, Orange, Connecticut; 2 Yale Center for Analytic Sciences, Yale School of Public Health, New Haven, Connecticut; 3 Pain Research, Informatics, Multi-morbidities, and Education Center, VA Connecticut Healthcare System, West Haven; 4 Department of Neurology, Yale School of Medicine, New Haven, Connecticut; 5 Headache Center of Excellence, VA Minneapolis Health Care System, Minneapolis, Minnesota; 6 Headache Center of Excellence, VA Connecticut Healthcare System, West Haven; 7 The Saul R. Korey Department of Neurology, Albert Einstein College of Medicine, Bronx, New York; 8 Ferkauf Graduate School of Psychology, Yeshiva University, Bronx, New York; 9 Department of Internal Medicine, Yale School of Medicine, New Haven, Connecticut

## Abstract

**Importance:**

Calcitonin gene–related peptide (CGRP), a neuropeptide involved in migraine pathophysiology, is also a key neuroimmune modulator. CGRP antagonists may help mitigate the hyperinflammatory response observed in patients with COVID-19; however, findings from the literature are contradictory, and to date, no study has investigated the safety and effectiveness of CGRP antagonists against COVID-19.

**Objective:**

To evaluate the association between CGRP monoclonal antibody (mAb) treatment and risk of SARS-CoV-2 infection and sequela hospitalization, requiring supplemental oxygen, use of mechanical ventilation, or death.

**Design, Setting, and Participants:**

This retrospective cohort study analyzed the electronic health records of US veterans aged 18 to 65 years who were diagnosed with migraine disorder and were at risk of COVID-19 between January 20, 2020, and May 19, 2022.

**Exposure:**

Initiation of CGRP mAbs.

**Main Outcomes and Measures:**

The main outcome was cumulative incidence of SARS-CoV-2 infection. Odds of 30-day hospitalization, requiring supplemental oxygen, use of mechanical ventilation, or death were secondary outcomes.

**Results:**

Among 8 178 652 eligible person-trials (354 294 veterans), 9992 (mean [SD] age, 46.0 [9.5] years; 53.9% male) initiated CGRP mAbs and 8 168 660 (mean [SD] age, 46.6 [10.2] years; 65.7% male) did not initiate CGRP mAbs. Over a 28-month follow-up period, 1247 initiators (12.5%) and 780 575 noninitiators (9.6%) tested positive for SARS-CoV-2. After censoring persons who deviated from treatment, the incidence was 7.4 cases per 1000 person-months among initiators and 6.9 per 1000 person-months among noninitiators. The inverse probability–weighted observational analogs of intention-to-treat and per-protocol hazard ratios were 0.95 (95% CI, 0.89-1.01) and 0.93 (95% CI, 0.86-1.02), respectively. No significant differences in the likelihood of hospitalization (odds ratio [OR], 0.93; 95% CI, 0.62-1.41), requiring supplemental oxygen (OR, 0.77; 95% CI, 0.45-1.30), use of mechanical ventilation (OR, 0.85; 95% CI, 0.26-2.84), or death (OR, 0.67; 95% CI, 0.09-5.23) were observed between CGRP mAb initiators and noninitiators who tested positive for SARS-CoV-2.

**Conclusions and Relevance:**

In this cohort study, CGRP mAb treatment was not associated with positive SARS-CoV-2 test results or risk of severe COVID-19 outcomes, suggesting that CGRP mAbs may be used for migraine prevention during the COVID-19 pandemic. Given the few events of requiring supplemental oxygen, use of mechanical ventilation, and death, replication analysis in a larger sample of patients later in the course of disease is warranted.

## Key Points

**Question:**

Is there an association of calcitonin gene–related peptide (CGRP) monoclonal antibody (mAb) use with risk of SARS-CoV-2 infection and developing severe clinical outcomes?

**Findings:**

In this cohort study of 354 294 US veterans with migraine disorder (8 178 652 person-trials), CGRP mAb treatment was not associated with a significant increase or reduction in risk of SARS-CoV-2 infection, hospitalization, requiring supplemental oxygen, use of mechanical ventilation, or death.

**Meaning:**

These findings suggest that CGRP mAbs may be used as a prophylaxis for patients with migraine who are at risk of or have COVID-19.

## Teaser

This cohort study assesses the association of calcitonin gene-related peptide (CGRP) monoclonal antibody (mAb) treatment with risk of SARS-CoV-2 infection among US veterans with migraine disorder.

## Introduction

Calcitonin gene–related peptide (CGRP) is a potent, vasodilated neuropeptide involved in migraine pathophysiology.^1^ CGRP antagonists and monoclonal antibodies (mAbs) targeting CGRP have been associated with aborting and preventing migraine attacks.^2,3^ CGRP has also been found in pulmonary afferent nerve fibers and may play a role in broncho-protection and immunomodulation.^4^ In vitro studies have observed that CGRP enhances the production of proinflammatory cytokines including interleukin 1 (IL-1) and IL-6 and antagonizes the production of anti-inflammatory cytokines such as IL-13.^5,6^ The literature has reported that CGRP attributed to smoke inhalation or acid-induced lung injury by exacerbating airway hyperemia, microvascular hyperpermeability, mucus secretion and bronchoconstriction in animal models, and disruption of calcitonin-related polypeptide alpha expression attenuated the respiratory failure.^7,8^ Given the immunomodulatory property, there is a hypothesis that CGRP antagonists might help mitigate the hyperinflammatory response during SARS-CoV-2 infection.

Cytokine storm is implicated in severe COVID-19, leading to acute respiratory distress syndrome and septic shock.^9^ The upregulation of receptor activity modifying protein 1, believed to be the receptor of CGRP, has been found in lung tissues of patients with COVID-19 who died.^10^ Patients who underwent lung transplant, which completely denervated the graft, had worse outcomes compared with patients who did not undergo lung transplant despite a similar clinical presentation of COVID-19.^11^ However, there is no clinical evidence to support the use of CGRP mAbs or antagonists for COVID-19 treatment. Preliminary studies on the effect of CGRP in sepsis or COVID-19 are inconsistent. A blinded randomized trial in a porcine model found that olcegepant was not beneficial in polymicrobial sepsis.^12^ A cross-sectional study reported that CGRP levels were decreased in 57 patients with COVID-19 and suggested that restoration of normal CGRP circulatory levels can be therapeutic.^13^ On the contrary, another prospective cohort study observed that higher plasma CGRP levels were associated with a higher risk of hospitalization and pulmonary intravascular coagulopathy, highlighting a role for plasma CGRP in sustaining COVID-19–related hyperinflammation.^14^ Furthermore, a multicenter study did not find significant differences in COVID-19 prevalence or disease course among 300 patients with migraine who received or did not receive CGRP mAbs.^15^ To address this knowledge gap, we used data from the Veterans Health Administration (VHA), the largest integrated health system in the US, and emulated a target trial to examine the association of CGRP mAbs with risk of SARS-CoV-2 infection and developing severe clinical outcomes among US veterans who were diagnosed with migraine disorder between January 20, 2020, and May 19, 2022.

## Methods

### Specification and Emulation of the Target Trial

We designed this retrospective cohort study to emulate a target trial of CGRP mAbs vs placebo among US veterans who were diagnosed with migraine disorder in the VHA. Table 1 summarizes the key protocol components of the target trial. This study was approved by the Research & Development Committee of the VA Connecticut Healthcare System and was granted a waiver of informed consent because it posed minimal risk to privacy and could not be carried out without a waiver due to its retrospective and large-scale nature. The study followed the Strengthening the Reporting of Observational Studies in Epidemiology (STROBE) reporting guideline for cohort studies.

**Table 1.** Specification and Emulation of a Target Trial Evaluating CGRP mAb Treatment and Risk of SARS-CoV-2 Infection and Severe Outcomes in the VHA

Protocol component	Target trial specification	Target trial emulation
Eligibility criteria	Aged 18-65 y with a 12-mo history of migraine, no previous SARS-CoV-2 infection, no contraindication or recent prescription of CGRP mAb (>5 mo), no prior use of CGRP receptor antagonists (rimegepant, ubrogepant, or atogepant), regular health care system contact, potential follow-up, not residing in long-term care facilities, and baseline defined as the first eligibility month	In addition to the target trial specification, regular contact defined as having an assigned VHA primary care practitioner at baseline, any outpatient or inpatient encounter in the past 12 mo, and potential follow-up defined as any outpatient or inpatient encounters during the study period
Treatment strategies	Initiation of CGRP mAb at baseline and continued use until a contraindication and no initiation of CGRP mAb during follow-up unless an indication developed	In addition to the target trial specification, discontinuation defined as a 30-d or longer gap between successive prescriptions
Treatment assignment	Randomly assigned and aware of assigned strategy at baseline	Classified individuals based on their compatible data at baseline, adjusted for baseline confounders to emulate target trial
Outcome	Primary outcome: PCR-confirmed SARS-CoV-2 infection; secondary outcomes: hospitalization, hospitalization with supplemental oxygen, hospitalization with invasive mechanical ventilation, and death, all within 30 d of positive test result	Same as for the target trial specification
Follow-up	Started at baseline and ended at the earliest occurrence of first PCR-confirmed SARS-CoV-2 infection, death, loss of follow-up, or administrative end of follow-up (May 19, 2022)	In addition to the target trial specification, loss of follow-up defined as the last VHA visit
Causal contrasts	Intention-to-treat outcome or per-protocol outcome	Observational analog of intention-to-treat outcome and per-protocol outcome
Statistical analysis	Intention-to-treat analysis: analyzed based on assigned treatment regardless of adherence; per-protocol analysis: accounted for deviations, adjusted for baseline and time-varying confounders using weights	Same intention-to-treat and per-protocol analyses with sequential emulation and additional adjustment for baseline covariates

Abbreviations: CGRP, calcitonin gene–related peptide; mAb, monoclonal antibody; PCR, polymerase chain reaction; VHA, Veterans Health Administration.

We identified eligible individuals from the VHA Headache Centers of Excellence administrative data cohort. This cohort includes 1.9 million veterans who were diagnosed with headache disorders since October 2007 and characterizes demographics, comorbidities, headache-related health care utilization, and treatments.^16^ The eligibility criteria were the same as those described for the target trial. To minimize measurement errors, we restricted the study sample to individuals in regular contact with the health care system, as prescriptions and hospitalizations outside the VHA were not captured. Individuals who had serious hypersensitivity to CGRP mAbs were rare and difficult to identify using administrative data. Therefore, we assumed that receiving CGRP mAbs indicated that there was a determination of no hypersensitivity. CGRP mAb prescriptions were identified using outpatient pharmacy records for erenumab, fremanezumab (225 mg/1.5 mL), and galcanezumab (120 mg/mL). We searched for eptinezumab but found no records of dispense. Individuals with a concurrent cluster headache and receiving galcanezumab, 100 mg/mL, were excluded from the study. Next, we classified eligible individuals according to the strategy of compatibility with their data at baseline. We assumed groups were exchangeable conditional on baseline demographics (age, gender, self-reported race [Black, White, and other or unknown; other included Alaska Native or American Indian, Asian, multiracial, or Native Hawaiian or other Pacific Islander], self-reported ethnicity [Hispanic], insurance status, VHA service connection, smoking status, and body mass index), care assessment need score,^17^ migraine characteristics (chronic migraine; headache-related primary care, emergency department, and neurology visits; use of triptans, anticonvulsants, angiotensin-converting enzyme inhibitors or angiotensin II receptor blockers, β-blockers, tricyclic antidepressants, serotonin and norepinephrine reuptake inhibitors, or neurotoxins; and number of migraine prophylactic classes^18^), comorbidities (hypertension, coronary artery disease, peripheral vascular disease, ischemic stroke or transient ischemic attack, chronic obstructive pulmonary disease, diabetes, chronic kidney disease, congestive heart failure, immunocompromised status, and Charlson Comorbidity Index), and number of COVID-19 vaccine doses (eTable 1 in Supplement 1). Race and ethnicity were included in the analysis to reduce the number of unknown data and to adjust them in the models to provide a more accurate estimation because they are associated with the likelihood of initiating CGRP mAb and COVID-19 outcomes. Missing values of categorical variables were coded as unknown.

The primary outcome was cumulative incidence of SARS-CoV-2 infection. The occurrences of SARS-CoV-2 infection were obtained from the Veterans Affairs (VA) COVID-19 Shared Data Resource, which includes analytical variables provisioned by the VA Informatics and Computing Infrastructure on all VHA enrollees who had a polymerase chain reaction test positive for SARS-CoV-2 within the VHA system or those who tested positive outside the VHA but were documented in the VHA records. COVID-19–related clinical outcomes (hospitalization, requiring supplemental oxygen, use of mechanical ventilation, or death) within 30 days of an index positive SARS-CoV-2 test result, COVID-19–related symptoms within 30 days prior to the index date, and inflammatory markers within 7 days after the index date were extracted from the VA COVID-19 Shared Data Resource for secondary and exploratory analyses. We defined the date of CGRP mAb initiation to be the first date of a prescription release from the pharmacy and calculated the discontinuation date using the monthly dose and quantity of autoinjectors in each prescription. We considered treatment to be continuous if there was a gap of less than 30 days following the end of the days’ supply of the last fill. Each individual was followed up from baseline until the month of first COVID-19 infection, death, loss of follow-up (defined as the last date of a VHA encounter in October 2022), or administrative end of follow-up (May 19, 2022), whichever happened first. The emulated target trial was a series of trials starting at each of the 28 months between January 20, 2020, and May 19, 2022. Each individual may have met the eligibility criteria several times over the follow-up period and participated in multiple trials.

### Statistical Analysis

For statistical efficiency, we estimated the association of CGRP mAb treatment with risk of SARS-CoV-2 infection by pooling data across all eligible person-trials. The intention-to-treat analysis examined the association of initiation of CGRP mAbs at baseline with risk of SARS-CoV-2 infection. To estimate the observational analog, we fitted a pooled logistic regression model including an indicator of CGRP mAb initiation and baseline confounders. We assumed that there were no unmeasured confounders. Given the covariates included in the model and a low monthly incidence of SARS-CoV-2 infection within levels of those covariates, the exponentiated coefficient for the CGRP mAb initiator approximated the intention-to-treat hazard ratio (HR). Of interest, the per-protocol analysis examined the association of adherence to the assigned treatment strategy throughout follow-up, which required additional adjustment for potential selection bias due to loss of follow-up. Estimation of the per-protocol outcome needed to be adjusted for potential selection bias due to loss of follow-up. First, we censored individuals at the month when they discontinued CGRP mAb treatment in the initiator group and censored individuals at the month when they initiated CGRP mAbs in the noninitiator group. Next, we fitted the same pooled logistic regression model as for the intention-to-treat analysis with this censored data, with stabilized inverse probability of treatment weighting (IPTW) applied to adjust for time-varying confounders (eTable 1 in Supplement 1).

Informally, the numerator of the IPTW was the probability that an individual received their observed treatment conditional on their treatment history and baseline confounders, and the denominator was the probability that an individual received their observed treatment given their treatment and covariate history (baseline and most recent covariates). We estimated the weight by fitting 2 separate models to allow the probability to differ according to prior treatment status among (1) person-trials that included participants who were untreated in the previous month or (2) person-trials that included participants who were treated in the previous month. Because we allowed a 30-day gap, the probability of receiving treatment for the month after treatment initiation was equal to 1. Individuals stopped contributing to the weight models once they deviated from their assigned treatment strategy. We evaluated different covariate structures to select the best-fitted model that produced weights with smaller variance. A mean estimated weight far from 1 or the presence of extreme values was considered as a model misspecification or violation of the positivity assumption.^19^ Then we truncated the stabilized IPTWs at their 99.9th percentile to increase the precision when assessing the treatment effect. The final weight for each individual at each time point was the product of the weights for that individual until that time and created a hypothetical population^20^ in which treatment was independent of the measured confounders at all time points.

Secondary analysis of the clinical outcomes was performed among patients with a positive SARS-CoV-2 test result. Weighted logistic regression was used to estimate the odds of hospitalization, requiring supplemental oxygen, use of mechanical ventilation, or death with adjustment of baseline covariates. To address the cluster effect due to replications, robust sandwich-type variance estimation was used to correct the SEs. Preindex symptoms among unique individuals were displayed as counts and percentages, and the difference between initiators and noninitiators were compared using χ^2^ tests. Inflammatory markers were displayed as medians and IQRs and compared using a Wilcoxon rank sum test. *P* values were adjusted using the Bonferroni procedure for multiple comparisons. All statistical analyses were performed using R, version 4.1.3 (R Foundation for Statistical Computing). A 2-tailed *P* value <.05 was used to indicate statistical significance.

## Results

The Figure shows the flowchart of patient selection for emulating the target trial. There were 354 294 unique veterans who contributed 8 178 652 eligible person-trials. Table 2 shows the baseline characteristics of 9992 person-trials who initiated CGRP mAb treatment (mean [SD] age, 46.0 [9.5] years; 53.9% men; 46.1% women) and 8 168 660 person-trials who did not initiate CGRP mAbs (mean [SD] age, 46.6 [10.2] years; 65.7% men; 34.3% women). Of the participants who initiated CGRP mAb treatment, 2593 (26.0%) were Black, 1240 (12.4%) were Hispanic, 6330 (63.4%) were White, and 1069 (10.7%) reported other or unknown race. Of the participants who did not initiate CGRP mAb treatment, 2 224 616 (27.2%) were Black, 822 093 (10.1%) were Hispanic, 5 051 692 (61.8%) were White, and 892 352 (10.9%) reported other or unknown race and ethnicity. Compared with noninitiators at baseline, CGRP mAb initiators included a higher proportion of women and persons who lived in urban areas, never smoked, were diagnosed with chronic migraine, had depression or were immunocompromised, and were prescribed triptans and migraine prophylaxis, such as anticonvulsants, β-blockers, tricyclic antidepressants, serotonin and norepinephrine reuptake inhibitors, and neurotoxins. Initiators had higher health care utilization in primary care, emergency department, and neurology due to their headache and a higher number of SARS-CoV-2 tests (1.2 [3.3%] vs 0.7 [2.5%]).

**Figure.**
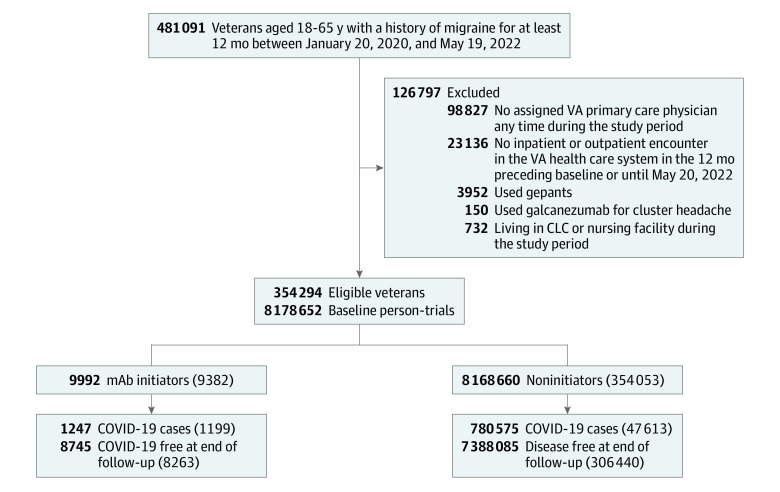
Flowchart of Eligible Veterans for Emulating a Target Trial of Calcitonin Gene–Related Peptide Monoclonal Antibody and Risk of SARS-CoV-2 Infection and Severe Outcomes From January 20, 2020, to May 19, 2022 Numbers in parentheses represent unique individuals in each group. The presented counts of initiators and noninitiators do not sum to the total number of eligible individuals because some eligible individuals contributed to both groups in different nested emulated trials. CLC indicates community living center; VA, Veterans Affairs.

**Table 2.** Baseline Characteristics of Eligible Person-Trials in the Emulated Target Trial of Calcitonin Gene–Related Peptide Monoclonal Antibody Treatment and Risk of SARS-CoV-2 Infection and Severe Outcomes in the VHA

Characteristic	Person-trials, No. (%)^a^	
	Initiators (n = 9992)^b^	Noninitiators (n = 8 168 660)^b^
Age, mean (SD), y	46.0 (9.5)	46.6 (10.2)
Gender		
Men	5384 (53.9)	5 367 058 (65.7)
Women	4608 (46.1)	2 801 602 (34.3)
Race		
Black	2593 (26.0)	2 224 616 (27.2)
White	6330 (63.4)	5 051 692 (61.8)
Other or unknown^c^	1069 (10.7)	892 352 (10.9)
Hispanic ethnicity	1240 (12.4)	822 093 (10.1)
Marital status		
Married	5514 (55.2)	4 267 936 (52.2)
Single	1598 (16.0)	1 495 777 (18.3)
Divorced, separated, or widowed	2797 (28.0)	2 309 170 (28.3)
Unknown	83 (0.8)	95 777 (1.2)
Rurality		
Urban	7363 (73.7)	5 702 039 (69.8)
Rural	2623 (26.3)	2 453 289 (30.0)
Unknown	6 (0.1)	13 332 (0.2)
VHA service connection	9642 (96.5)	7 491 849 (91.7)
Insurance status		
Insured	4628 (46.3)	3 534 787 (43.3)
Not insured	5333 (53.4)	4 610 269 (56.4)
Unknown	31 (0.3)	23 604 (0.3)
Smoking status		
Never	5193 (52.0)	3 621 104 (44.3)
Current	3104 (31.1)	3 133 711 (38.4)
Former	1694 (17.0)	1 411 194 (17.3)
Unknown	1 (<0.1)	2651 (<0.1)
Body mass index^d^		
Mean (SD)	31.3 (6.1)	31.1 (6.1)
Underweight or normal	1350 (13.5)	1 137 501 (13.9)
Overweight	3005 (30.1)	2 489 543 (30.5)
Obesity I	3008 (30.1)	2 360 847 (28.9)
Obesity II	1583 (15.8)	1 204 765 (14.7)
Obesity III	812 (8.1)	631 670 (7.7)
Unknown	234 (2.3)	344 334 (4.2)
Migraine duration, mean (SD), y	6.2 (3.9)	6.0 (3.8)
Chronic migraine	2963 (29.7)	573 582 (7.0)
Headache-related visits in the past year, mean (SD)		
Primary care	1.2 (1.3)	0.6 (0.9)
Emergency department	0.2 (1.2)	0.0 (0.3)
Neurology	2.0 (2.1)	0.2 (0.9)
Prescribed triptans	8366 (83.7)	4 776 138 (58.5)
Prophylactic medications^e^		
Mean (SD), No.	2.5 (1.3)	1.2 (1.2)
Anticonvulsants	7326 (73.3)	3 024 382 (37.0)
ACEIs or ARBs	1449 (14.5)	1 248 531 (15.3)
β-Blockers	4651 (46.5)	2 141 949 (26.2)
Tricyclic antidepressants	3805 (38.1)	1 451 448 (17.8)
SNRIs	2315 (23.2)	1 159 301 (14.2)
Neurotoxins	4459 (44.6)	385 704 (4.7)
Comorbidities		
Chronic obstructive pulmonary disease	435 (4.4)	384 729 (4.7)
Hypertension	3525 (35.3)	3 013 135 (36.9)
Congestive heart failure	142 (1.4)	136 517 (1.7)
Coronary artery disease	413 (4.1)	360 147 (4.4)
Transient ischemic attack	93 (0.9)	55 559 (0.7)
Ischemic stroke	148 (1.5)	97 546 (1.2)
Peripheral vascular disease	133 (1.3)	143 310 (1.8)
Diabetes	1350 (13.5)	1 150 761 (14.1)
Chronic kidney disease	348 (3.5)	250 032 (3.1)
Immunocompromised status^f^	679 (6.8)	367 092 (4.5)
Depression	6617 (66.2)	4 368 086 (53.5)
Charlson Comorbidity Index score, mean (SD)	0.8 (1.4)	0.7 (1.4)
Care assessment need score, mean (SD)	17.0 (17.1)	20.2 (18.3)
COVID-19 vaccine doses		
Mean (SD), No.	0.5 (0.9)	0.6 (1.0)
0	7408 (74.1)	5 791 189 (70.8)
1	394 (3.9)	401 944 (4.9)
2	1653 (16.5)	1 552 919 (19.0)
3	537 (5.4)	432 608 (5.3)
SARS-CoV-2 tests during follow-up, mean (SD), No.	1.2 (3.3)	0.7 (2.5)

Abbreviations: ACEIs, angiotensin-converting enzyme inhibitors; ARBs, angiotensin II receptor blockers; SNRIs, serotonin and norepinephrine reuptake inhibitors; VHA, Veterans Health Administration.

^a^ Percentages may not total 100% due to rounding. Data are reported as number (percentage) of person-trials unless otherwise indicated.

^b^ Each individual may have contributed to more than 1 trial.

^c^ Other included Alaska Native or American Indian, Asian, Native Hawaiian or other Pacific Islander, and multiracial.

^d^ Calculated as weight in kilograms divided by height in meters squared. Obesity class I is defined as a body mass index of 30 to 35; class II, 36 to 40; and class III, 41 or higher.

^e^ Migraine prophylactic medications were classified per the Department of Veterans Affairs and the Department of Defense Clinical Practice Guideline for the Primary Care Management of Headache^18^ as anticonvulsants (lamotrigine, pregabalin, topiramate, and valproates), ACEIs or ARBs (lisinopril and candesartan), β-blockers (atenolol, bisoprolol, metoprolol, nadolol, propranolol, and timolol), tricyclic antidepressants (amitriptyline and nortriptyline), SNRIs (venlafaxine), and neurotoxins (abobotulinumtoxinA, incobotulinumtoxinA, and onabotulinumtoxinA).

^f^ Includes diagnosis of HIV infection, organ or tissue transplant, bone marrow biopsy, or use (prescribed ≥2 times over the past year) of systematic glucocorticoids, antirheumatic agents in combination with glucocorticoids, or immunosuppressants.

Table 3 shows the estimated HRs of SARS-CoV-2 infection and odds ratios (ORs) of developing severe clinical outcomes when comparing CGRP mAb initiators with noninitiators. A total of 1247 initiators (12.5%) and 780 575 noninitiators (9.6%) tested positive for SARS-CoV-2 from their baseline until May 19, 2022 (eTable 2 in Supplement 1). A lower proportion of initiators were hospitalized (55 [4.4%] vs 37 724 [4.8%]), required supplemental oxygen (36 [2.9%] vs 26 153 [3.4%]), or died (2 [0.2%] vs 3594 [0.5%]) within 30 days after the positive test result. The estimated observational analog of the intention-to-treat HR was 0.95 (95% CI, 0.89-1.01), indicating no significant difference between initiators and noninitiators. There were also no significant differences between initiators and noninitiators for hospitalization (OR, 0.89; 95% CI, 0.66-1.20), requiring supplemental oxygen (OR, 0.81; 95% CI, 0.57-1.17), or death (OR, 0.53; 95% CI, 0.13-2.13) after adjusting for baseline confounders.

**Table 3.** Cumulative Incidence of SARS-CoV-2 Infection and Likelihood of Developing Severe Outcomes Among Calcitonin Gene–Related Peptide Monoclonal Antibody Initiators and Noninitiators

Analysis	Events/person-trials, No. (%)		HR or OR (95% CI)^a^	*P* value
	Initiators	Noninitiators		
**Intention-to-treat**				
SARS-CoV-2 infection	1247/9992 (12.5)	780 575/8 168 660 (9.6)	0.95 (0.89-1.01)	.08
Hospitalization	55/1247 (4.4)	37 724/780 575 (4.8)	0.89 (0.66-1.20)	.45
Supplemental oxygen	36/1247 (2.9)	26 153/780 575 (3.4)	0.81 (0.57-1.17)	.27
Mechanical ventilation	8/1247 (0.6)	4715/780 575 (0.6)	1.02 (0.50-2.09)	.96
Death	2/1247 (0.2)	3594/780 575 (0.5)	0.53 (0.13-2.13)	.37
**Per-protocol**				
SARS-CoV-2 infection	565/9992 (5.7)	771 215/8 168 660 (9.4)	0.93 (0.86-1.02)	.12
Hospitalization	27/565 (4.8)	37 425/771 215 (4.9)	0.93 (0.62-1.41)	.75
Supplemental oxygen	16/565 (2.8)	25 950/771 215 (3.4)	0.77 (0.45-1.30)	.32
Mechanical ventilation	3/565 (0.5)	4668/771 215 (0.6)	0.85 (0.26-2.84)	.79
Death	1/565 (0.2)	3582/771 215 (0.5)	0.67 (0.09-5.23)	.70

Abbreviations: HR, hazard ratio; OR, odds ratio.

^a^ Cumulative incidence of SARS-CoV-2 infection is shown as estimated HRs; likelihood of developing severe outcomes is shown as ORs. Analyses were adjusted for baseline characteristics of age; gender; race; ethnicity; rurality; insurance status; Veterans Health Administration service connection; smoking status; body mass index; chronic migraine; headache-related primary care, emergency department, or neurology visits in the past year; prescribed triptans, anticonvulsants, angiotensin-converting enzyme inhibitors or angiotensin II receptor blockers, β-blockers, or tricyclic antidepressants or neurotoxins; number of prophylactic classes; hypertension; coronary artery disease; peripheral vascular disease; ischemic stroke or transient ischemic attack; chronic obstructive pulmonary disease; diabetes; chronic kidney disease; congestive heart failure; immunocompromised status; Charlson Comorbidity Index; care assessment need score; and number of COVID-19 vaccine doses. SEs were corrected using a robust sandwich-type variance estimation.

After censoring persons who deviated from their assigned treatment, 565 initiators (5.7%) and 771 215 noninitiators (9.4%) tested positive for SARS-CoV-2. The median duration of initiators receiving CGRP mAbs was 5 months (IQR, 2-11 months). The incidence of SARS-CoV-2 infection was 7.4 per 1000 person-months among initiators and 6.9 per 1000 person-months among noninitiators. The mean of the stabilized weight was 1.000 and ranged from 0.007 to 50.429. Details of the weight estimation are described in eTables 3 and 4 and the eFigure in Supplement 1. After truncating, the stabilized weight ranged from 0.950 to 1.153. The estimated observational analog of per-protocol outcomes using weighted models showed that risks of SARS-CoV-2 infection (HR, 0.93; 95% CI, 0.86-1.02), sequela hospitalization (OR 0.93; 95% CI, 0.62-1.41), requiring supplemental oxygen (OR, 0.77; 95% CI, 0.45-1.30), use of mechanical ventilation (OR, 0.85; 95% CI, 0.26-2.84), or death (OR, 0.67; 95% CI, 0.09-5.23) were not significantly different between initiators and noninitiators.

Table 4 shows preindex symptoms and inflammatory markers among 564 unique patients who received CGRP mAbs and 46 674 unique patients who did not initiate CGRP mAbs. Initiators were more likely to experience COVID-19–related symptoms (354 [62.8%] vs 26 297 [56.3%]; *P* = .04), such as abdominal pain (46 [8.2%] vs 2221 [4.8%]; *P* = .004) and sore throat (102 [18.1%] vs 6238 [13.4%]; *P* = .02). There were no statistically significant differences in dyspnea between initiators and noninitiators (154 [27.3%] vs 10 975 [23.5%]; *P* = .59). During the first 7 days after a positive test result, the median C-reactive protein levels were 2.3 mg/dL (IQR, 0.8-6.9 mg/dL) among initiators and 2.5 mg/dL (IQR, 0.7-7.2 mg/dL) among noninitiators (*P* = .92; to convert CRP to milligrams per liter, multiply by 10), which was not a significant difference. However, initiators had significantly lower ferritin (median, 148.0 ng/mL [IQR, 77.0-279.0 ng/mL] vs 290.0 ng/mL [IQR, 12.0-650.5 ng/mL]; *P* = .05; to convert to μg/L, multiply by 1.0) and procalcitonin (median, 0.05 ng/mL [IQR, 0.05-0.06 ng/mL] vs 0.07 ng/mL [IQR, 0.05-0.15 ng/mL]; *P* = .04) levels than noninitiators.

**Table 4.** COVID-19–Related Symptoms Present on the Index Date or Within the Preceding 30 Days Among Unique Veterans Who Tested Positive for SARS-CoV-2

Symptom	Veterans, No. (%)		*P* value^a^
	Initiators (n = 564)	Noninitiators (n = 46 674)	
Any	354 (62.8)	26 297 (56.3)	.04
Abdominal pain	46 (8.2)	2221 (4.8)	.004
Chills	65 (11.5)	4963 (10.6)	>.99
Cough	219 (38.8)	15 968 (34.2)	.36
Diarrhea	92 (16.3)	6354 (13.6)	>.99
Dyspnea	154 (27.3)	10 975 (23.5)	.59
Fatigue	98 (17.4)	6355 (13.6)	.17
Fever	159 (28.2)	12 227 (26.2)	>.99
Headache	157 (27.8)	10 829 (23.2)	.17
Loss of smell	20 (3.5)	1798 (3.9)	>.99
Loss of taste	56 (9.9)	3814 (8.2)	>.99
Myalgia	71 (12.6)	5340 (11.4)	>.99
Nausea	94 (16.7)	6153 (13.2)	.27
Rhinorrhea	56 (9.9)	4264 (9.1)	>.99
Sore throat	102 (18.1)	6238 (13.4)	.02
CRP level, median (IQR), mg/dL^b^	2.3 (0.8-6.9)	2.5 (0.7-7.2)	.92^c^
Ferritin level, median (IQR), ng/mL^d^	148.0 (77.0-279.0)	290.0 (12.0-650.5)	.05^c^
Procalcitonin level, median (IQR), ng/mL^e^	0.05 (0.05-0.06)	0.07 (0.05-0.15)	.04^c^

Abbreviation: CRP, C-reactive protein.

SI conversions: To convert CRP to milligrams per liter, multiply by 10; convert ferritin to micrograms per liter, multiply by 1.0.

^a^ *P* values were adjusted for multiple comparisons using the Bonferroni procedure.

^b^ Data were available for 34 (missing for 530) in the initiator group and 2140 (missing for 44 534) in the noninitiator group.

^c^ Wilcoxon rank sum test.

^d^ Data were available for 25 (missing for 539) in the initiator group and 1988 (missing for 44 686) in the noninitator group.

^e^ Data were available for 27 (missing for 537) in the initiator group and 1662 (missing for 45 012) in the noninitator group.

## Discussion

In this cohort study, we emulated a target trial to evaluate the association of CGRP mAb treatment with SARS-CoV-2 infection and severe clinical outcomes among veterans in the largest integrated health care system in the US. Treatment with CGRP mAb was not associated with risk of SARS-CoV-2 infection. Among those who tested positive for SARS-CoV-2, there were no significant differences in hospitalization, oxygen supplementation, mechanical ventilation, or COVID-19–related death between CGRP mAb initiators and noninitiators.

Although there is little biological reason to be concerned that CGRP antagonists may predispose patients to greater risk of contracting COVID-19, there is limited clinical evidence on the safety of prescribing CGRP mAbs for patients with migraine at risk of COVID-19. A multicenter study conducted between May and November 2020 found that the proportion of COVID-19 cases among patients treated with CGRP mAbs was 16.1%, which was not significantly different from the 11% among patients not treated with mAbs.^15^ However, the ascertainment of cases in that study was mostly based on self-reported symptoms, which may have led to a biased conclusion. In a 24-week clinical trial of eptinezumab for migraine prevention, the incidence of COVID-19, recorded as treatment-emergent adverse events, was also similar between treatment (6.2%) and placebo (5.4%) groups.^21^ Our study did not find a significant difference in the incidence of COVID-19 between patients receiving or not receiving CGRP mAbs (7.4 vs 6.9 cases per 1000 person-months), suggesting that CGRP mAbs may be used among patients who are at risk of or have COVID-19.

The discussion about repurposing CGRP antagonists to treat COVID-19 was based on findings that CGRP can enhance IL-6 production in vitro.^22^ Raised IL-6 levels and cytokine storm are associated with severe clinical outcomes in patients hospitalized with COVID-19, and administration of IL-6 antagonists is associated with reduced 28-day all-cause mortality.^23,24,25^ Yet, there is no study, to our knowledge, that directly observed the efficacy of CGRP antagonists for patients with COVID-19, and results from animal or observational studies are controversal.^13,14,26,27^ In our study, although we found that patients with migraine who were treated with CGRP mAbs had better inflammatory profiles (lower ferritin and procalcitonin levels), which could have been associated with better outcomes,^28^ their clinical courses were no better than controls in terms of hospitalization, requiring supplemental oxygen or mechanical ventilation, and death. However, those results should be interpreted with caution due to the small number of events in addition to the fact that CGRP induces inotropy, suppresses elevated pulmonary arterial pressure, and attenuates cardiac remodeling,^29,30^ which may be beneficial to patients with severe COVID-19 who develop acute respiratory distress and septic shock. The olcegepant trial concluded that the worse 72-hour survival rate observed in the treatment group could be explained by unstable hemodynamics.^12^ In addition, a recent meta-analysis found that patients hospitalized with COVID-19 had better survival if they experienced headache as a symptom.^31^ Considering the role of CGRP in migraine pathophysiology, the authors hypothesized that an increase in the CGRP level, which triggered headache as 1 of the COVID-19 symptoms, is a host compensatory response against COVID-19.

Of note, we found patients with COVID-19 who were treated with CGRP mAbs were more likely to report mild symptoms (abdominal pain and sore throat), but there were no significant differences in moderate symptoms such as dyspnea.^32^ One possible explanation is that receipt of a CGRP mAb is associated with a more positive health belief; 3 failures of conventional preventatives may not change patients’ perceptions so that they are more likely to react to cues when they experience COVID-19 symptoms. Patients treated with mAbs and their prescribers should be aware that milder symptoms of COVID-19 may present differently compared with symptoms in patients not treated with mAbs.

### Limitations

This study has several limitations. First, the median duration of CGRP mAb treatment was 5 months, which limited the ability to examine longer-term outcomes. Second, the conditional exchangeability assumption is untestable. However, we used expert knowledge to identify confounders associated with CGRP mAb initiation, explored various confounders and functional forms (eg, natural spline), and checked weight distribution for misspecifications to enhance the assumption’s plausibility. Third, the administrative data only captured patients with COVID-19 who tested positive for SARS-CoV-2 within or outside the VHA who had the case documented in the records. Prescriptions and hospitalizations outside the VHA were not captured. However, conducting this study within a national integrated health care system and up-to-standard VHA data minimized the misclassification bias. Additionally, CGRP mAb initiators tend to have higher levels of health care–seeking behaviors, reflected in more SARS-CoV-2 tests (1.2 vs 0.7), leading to overrepresentation and potentially a false-negative conclusion. Future studies should consider sensitivity analysis to explore residual confounding and mismeasurement.^33,34^ In addition, even though the study sample started with 354 294 eligible veterans, after conditioning on the probability of receiving CGRP mAbs and testing positive for SARS-CoV-2, the number of patients who required supplemental oxygen or mechanical ventilation or who died was small. Furthermore, the veterans in this sample were relatively young and healthy. The potential protective effects of CGRP antagonist may only occur among patients who are at high risk of developing severe COVID-19. However, considering the lower risk of progressing to severe COVID-19 with a less virulent variant, further investigation and collaborations are needed to fully understand the role of CGRP and its antagonist in the context of infection and the inflammatory process.

## Conclusions

In this cohort study on CGRP mAb treatment and COVID-19 outcomes in veterans with migraine, there was no significant difference in the incidence of COVID-19 or sequela hospitalization between CGRP mAb recipients and nonrecipients, suggesting that CGRP mAbs may be used for migraine prevention among patients who are at risk of or have COVID-19. However, there were few events of requiring supplemental oxygen, use of mechanical ventilation, and death among CGRP mAb initiators, indicating that replication analysis in a larger sample of patients later in the course of disease may be warranted.

## Notes

Supplement 1. **eTable 1.** Covariates Used When Emulating a Target Trial of CGRP mAb Treatment and Risk of SARS-CoV-2 Infection and Severe Outcomes **eTable 2.** Number of Participants, CGRP mAb Initiators, and COVID-19 Cases in Each Monthly Emulated Trial **eTable 3.** Odds Ratios and 95% Confidence Intervals From the Numerator and Denominator Models Estimating the Inverse Probability of Treatment Weights Among Person-Trials Who Were Untreated in the Previous Month **eTable 4.** Odds Ratios and 95% Confidence Intervals From the Numerator and Denominator Models Estimating the Inverse Probability of Treatment Weights Among Person-Trials Who Were Treated in the Previous Month **eFigure.** Distribution of Log-Transformed Stabilized Weight by Months

Supplement 2.
Data Sharing Statement
