# Supplementary material for: Calcitonin Gene–Related Peptide Monoclonal Antibodies and Risk of SARS-CoV-2 Infection and Severe COVID-19 Outcomes Among Veterans With Migraine Disorder
Source: JAMA Netw Open. 2023 Jul 31;6(7):e2326371. doi: 10.1001/jamanetworkopen.2023.26371 (PMC10391301; doi:10.1001/jamanetworkopen.2023.26371)
Supplement: Supplement 1. — eTable 1. Covariates Used When Emulating a Target Trial of CGRP mAb Treatment and Risk of SARS-CoV-2 Infection and Severe Outcomes eTable 2. Number of Participants, CGRP mAb Initiators, and COVID-19 Cases in Each Monthly Emulated Trial eTable 3. Odds Ratios and 95% Confidence Intervals From the Numerator and Denominator Models Estimating the Inverse Probability of Treatment Weights Among Person-Trials Who Were Untreated in the Previous Month eTable 4. Odds Ratios and 95% Confidence Intervals From the Numerator and Denominator Models Estimating the Inverse Probability of Treatment Weights Among Person-Trials Who Were Treated in the Previous Month eFigure. Distribution of Log-Transformed Stabilized Weight by Months [file jamanetwopen-e2326371-s001.pdf]

Supplemental Online Content

Wang K, Fenton BT, Deng Y, et al. Calcitonin gene–related peptide monoclonal antibodies and risk of SARS-CoV-2 infection and severe COVID-19 outcomes among veterans with migraine disorder. *JAMA Netw Open*. 2023;6(7):e2326371. doi:10.1001/jamanetworkopen.2023.26371

eTable 1. Covariates Used When Emulating a Target Trial of CGRP mAb Treatment and Risk of SARS-CoV-2 Infection and Severe Outcomes
eTable 2. Number of Participants, CGRP mAb Initiators, and COVID-19 Cases in Each Monthly Emulated Trial
eTable 3. Odds Ratios and 95% Confidence Intervals From the Numerator and Denominator Models Estimating the Inverse Probability of Treatment Weights Among Person-Trials Who Were Untreated in the Previous Month
eTable 4. Odds Ratios and 95% Confidence Intervals From the Numerator and Denominator Models Estimating the Inverse Probability of Treatment Weights Among Person-Trials Who Were Treated in the Previous Month
eFigure. Distribution of Log-Transformed Stabilized Weight by Months

This supplemental material has been provided by the authors to give readers additional information about their work.

© 2023 Wang K et al. *JAMA Network Open*.

eTable 1. Covariates Used When Emulating a Target Trial of CGRP mAb Treatment and Risk of SARS-CoV-2 Infection and Severe Outcomes

Covariates	Function form	Categories
**Time-fixed**		
Age	Natural spline (df=2)	
Gender	Binary	Men, Women
Race	Categorical	White, Black, Others/Unknown
Ethnicity	Binary	Hispanics, Non-Hispanics
Rurality	Categorical	Urban, Rural, Unknown
Insurance status	Categorical	Covered, Not covered, Unknown
VHA Service connection	Binary	Yes, No
Smoking status	Categorical	Never, Current, Former, Unknown
Body mass index	Categorical	Underweight/Normal, Overweight, Obese I, Obese II, Obese III, Unknown
Care assessment need score	Linear	
Chronic obstructive pulmonary disease	Binary	Yes, No
Diabetes mellitus	Binary	Yes, No
Chronic kidney disease	Binary	Yes, No
Congestive heart failure	Binary	Yes, No
Immunocompromised status	Binary	Yes, No
Number of COVID-19 vaccines	Linear	
Charlson comorbidity index	Linear	
Month of baseline	Natural spline (df=2)	
**Time-varying**		
Month of follow-up	Natural spline (df=2)	
Chronic migraine	Binary	Yes, No
Headache-related visits in the past year		
Primary care	Linear, log-transformed	
Emergency Department	Linear, log-transformed	
Neurology	Linear, log-transformed	
Ever Prescribed Triptans	Binary	Yes, No
Number of prophylactic classes	Linear	
Anticonvulsants	Binary	Yes, No
ACEIs/ARBs	Binary	Yes, No
β-blockers	Binary	Yes, No
Tricyclic antidepressants	Binary	Yes, No
Neurotoxins	Binary	Yes, No
Hypertension	Binary	Yes, No
Coronary artery disease	Binary	Yes, No
Peripheral vascular disease	Binary	Yes, No
Ischemic stroke/TIA	Binary	Yes, No
Depression	Binary	Yes, No

Abbreviations: df, degrees of freedom; ACEIs, angiotensin-converting enzyme inhibitors; ARBs, angiotensin II receptor blockers; TIA, transient ischemic attack.

© 2023 Wang K et al. *JAMA Network Open*.

eTable 2. Number of Participants, CGRP mAb Initiators, and COVID-19 Cases in Each Monthly Emulated Trial

Trial Number	Start Date	Participants	COVID-19 cases	Initiators	COVID-19 cases in initiators
1	20-Jan-2020	280,998	43,911	225	32
2	20-Feb-2020	283,304	44,272	212	38
3	20-Mar-2020	285,990	44,650	799	133
4	20-Apr-2020	287,463	44,549	1,019	192
5	20-May-2020	288,632	44,470	699	132
6	20-Jun-2020	289,949	44,397	425	86
7	20-Jul-2020	291,071	43,468	411	64
8	20-Aug-2020	292,590	42,889	319	54
9	20-Sep-2020	294,435	42,715	344	58
10	20-Oct-2020	296,696	42,404	348	69
11	20-Nov-2020	297,253	40,616	348	66
12	20-Dec-2020	296,112	37,772	277	40
13	20-Jan-2021	295,138	34,613	313	48
14	20-Feb-2021	295,201	33,029	354	48
15	20-Mar-2021	295,416	32,275	305	31
16	20-Apr-2021	295,657	31,371	293	49
17	20-May-2021	296,205	30,784	282	34
18	20-Jun-2021	297,157	30,599	264	24
19	20-Jul-2021	298,272	30,265	318	52
20	20-Aug-2021	297,034	27,894	257	31
21	20-Sep-2021	296,110	25,454	309	44
22	20-Oct-2021	296,490	24,129	290	39
23	20-Nov-2021	296,519	22,956	247	28
24	20-Dec-2021	295,924	21,228	205	17
25	20-Jan-2022	285,972	9,803	302	8
26	20-Feb-2022	283,410	6,079	237	6
27	20-Mar-2022	284,433	5,583	283	9
28	20-Apr-2022	285,221	5,167	307	9

© 2023 Wang K et al. *JAMA Network Open*.

eTable 3. Odds Ratios and 95% Confidence Intervals From the Numerator and Denominator Models Estimating the Inverse Probability of Treatment Weights Among Person-Trials Who Were Untreated in the Previous Month

	Numerator	Denominator
Baseline covariates		
Age, natural spline 1	1.14 (1.05, 1.23)	1.22 (1.13, 1.32)
Age, natural spline 2	0.69 (0.68, 0.71)	0.73 (0.71, 0.75)
Men vs. Women	0.71 (0.70, 0.72)	0.73 (0.72, 0.74)
Race, Black vs. White	0.82 (0.81, 0.83)	0.82 (0.81, 0.84)
Race, Others/Unknown vs. White	0.90 (0.88, 0.91)	0.89 (0.87, 0.91)
Hispanics vs. Non-Hispanics	0.95 (0.93, 0.97)	0.94 (0.92, 0.96)
Rurality, Rural vs. Urban	0.94 (0.93, 0.95)	0.95 (0.93, 0.96)
Rurality, Unknown vs. Urban	0.50 (0.40, 0.63)	0.54 (0.43, 0.67)
VHA Service connected, Yes vs. No	0.71 (0.68, 0.73)	0.73 (0.71, 0.75)
Insurance, Not Covered vs. Covered	0.91 (0.90, 0.92)	0.91 (0.90, 0.92)
Insurance, Unknown vs. Covered	1.05 (0.93, 1.18)	1.08 (0.96, 1.21)
Chronic migraine, Yes vs. No	1.12 (1.10, 1.14)	1.10 (1.08, 1.12)
Headache-related visits in the past year		
Primary Care, log-transformed	1.21 (1.21, 1.22)	1.20 (1.19, 1.20)
Emergency room, log-transformed	1.15 (1.14, 1.16)	1.14 (1.13, 1.15)
Neurology, log-transformed	1.57 (1.56, 1.58)	1.52 (1.51, 1.53)
Prescribed Triptans, Yes vs. No	1.53 (1.51, 1.56)	0.44 (0.42, 0.45)
Number of prophylactic classes	1.04 (1.03, 1.06)	0.75 (0.71, 0.79)
Anticonvulsants, Yes vs. No	2.00 (1.95, 2.04)	0.78 (0.73, 0.83)
ACEIs/ARBs, Yes vs. No	0.90 (0.88, 0.92)	1.03 (0.94, 1.12)
β-blockers, Yes vs. No	1.45 (1.42, 1.48)	0.73 (0.69, 0.78)
Tricyclic antidepressants, Yes vs. No	1.46 (1.43, 1.49)	0.81 (0.76, 0.87)
Neurotoxins, Yes vs. No	2.66 (2.60, 2.71)	1.04 (0.98, 1.10)
Hypertension, Yes vs. No	0.94 (0.92, 0.95)	0.99 (0.94, 1.04)
Peripheral vascular disease, Yes vs. No	0.64 (0.60, 0.68)	1.09 (0.92, 1.28)
Ischemic stroke /TIA, Yes vs. No	0.98 (0.94, 1.03)	1.07 (0.92, 1.25)
Coronary artery disease, Yes vs. No	0.96 (0.93, 0.99)	0.92 (0.83, 1.01)
Depression, Yes vs. No	1.06 (1.05, 1.08)	0.73 (0.70, 0.76)
Month of baseline, natural spline1	0.38 (0.37, 0.40)	0.39 (0.37, 0.40)
Month of baseline, natural spline 2	0.66 (0.64, 0.68)	0.66 (0.63, 0.68)
Time-varying covariates		
Month of follow-up, natural spline 1	0.34 (0.33, 0.35)	0.22 (0.21, 0.23)
Month of follow-up, natural spline 2	0.59 (0.57, 0.61)	0.44 (0.42, 0.45)
Prescribed Triptans, Yes vs. No		3.95 (3.79, 4.10)
Anticonvulsants, Yes vs. No		2.83 (2.67, 3.01)
ACEIs/ARBs, Yes vs. No		0.89 (0.82, 0.97)
β-blockers, Yes vs. No		2.09 (1.97, 2.22)
Tricyclic antidepressants, Yes vs. No		1.82 (1.71, 1.94)
Neurotoxins, Yes vs. No		2.87 (2.70, 3.04)
Number of prophylactic classes		1.39 (1.32, 1.45)
Hypertension, Yes vs. No		0.94 (0.89, 0.98)
Peripheral vascular disease, Yes vs. No		0.59 (0.50, 0.69)
Ischemic stroke/TIA, Yes vs. No		0.94 (0.81, 1.08)
Coronary artery disease, Yes vs. No		1.05 (0.96, 1.15)
Depression, Yes vs. No		1.46 (1.40, 1.52)

Abbreviations: ACEIs, angiotensin-converting enzyme inhibitors; ARBs, angiotensin II receptor blockers; TIA, transient ischemic attack.

© 2023 Wang K et al. *JAMA Network Open*.

eTable 4. Odds Ratios and 95% Confidence Intervals From the Numerator and Denominator Models Estimating the Inverse Probability of Treatment Weights Among Person-Trials Who Were Treated in the Previous Month

	Numerator	Denominator
Baseline covariates		
Age, natural spline 1	2.08 (1.59, 2.72)	2.08 (1.59, 2.71)
Age, natural spline 2	1.39 (1.24, 1.56)	1.39 (1.24, 1.56)
Men vs. Women	0.86 (0.82, 0.91)	0.86 (0.81, 0.91)
Race, Black vs. White	0.66 (0.62, 0.70)	0.65 (0.61, 0.70)
Race, Others/Unknown vs. White	0.8 (0.74, 0.88)	0.80 (0.73, 0.87)
Hispanics vs. Non-Hispanics	0.77 (0.71, 0.84)	0.77 (0.71, 0.84)
Rurality, Rural vs. Urban	1.12 (1.05, 1.20)	1.12 (1.05, 1.19)
Rurality, Unknown vs. Urban	1.78 (0.54, 5.88)	1.78 (0.54, 5.88)
VHA Service connection, Yes vs. No	0.99 (0.85, 1.15)	0.99 (0.85, 1.16)
Insurance, Not Covered vs. Covered	0.93 (0.88, 0.98)	0.93 (0.88, 0.98)
Insurance, Unknown vs. Covered	0.83 (0.51, 1.35)	0.82 (0.51, 1.32)
Chronic migraine, Yes vs. No	1.06 (1.00, 1.13)	1.06 (1.00, 1.13)
Headache-related visits in the past year		
Primary Care, log-transformed	1.03 (1.01, 1.05)	1.03 (1.01, 1.05)
Emergency room, log-transformed	1.04 (1.01, 1.08)	1.05 (1.01, 1.08)
Neurology, log-transformed	1.03 (1.01, 1.05)	1.03 (1.01, 1.05)
Prescribed Triptans, Yes vs. No	1.04 (0.97, 1.12)	1.13 (0.95, 1.35)
Number of prophylactic classes	0.99 (0.94, 1.05)	0.78 (0.58, 1.05)
Anticonvulsants, Yes vs. No	1.09 (1.00, 1.19)	1.09 (0.72, 1.65)
ACEIs/ARBs, Yes vs. No	0.84 (0.76, 0.94)	0.98 (0.62, 1.57)
β-blockers, Yes vs. No	1.13 (1.04, 1.22)	1.61 (1.10, 2.34)
Tricyclic antidepressants, Yes vs. No	1.12 (1.03, 1.21)	1.47 (0.96, 2.24)
Neurotoxins, Yes vs. No	0.99 (0.92, 1.08)	1.95 (1.24, 3.06)
Hypertension, Yes vs. No	1.05 (0.98, 1.12)	0.88 (0.67, 1.16)
Peripheral vascular disease, Yes vs. No	1.08 (0.85, 1.37)	1.34 (0.68, 2.64)
Ischemic stroke /TIA, Yes vs. No	0.88 (0.73, 1.06)	1.64 (0.82, 3.29)
Coronary artery disease, Yes vs. No	1.00 (0.86, 1.15)	1.50 (0.90, 2.49)
Depression, Yes vs. No	0.91 (0.86, 0.97)	0.84 (0.65, 1.09)
Month of baseline, natural spline1	0.98 (0.82, 1.17)	0.98 (0.82, 1.16)
Month of baseline, natural spline 2	0.82 (0.73, 0.92)	0.82 (0.73, 0.92)
Time-varying covariates		
Month of follow-up, natural spline 1	25.12 (21.64, 29.16)	24.99 (21.45, 29.11)
Month of follow-up, natural spline 2	1.56 (1.24, 1.97)	1.56 (1.24, 1.98)
Triptans, Yes vs. No		0.91 (0.75, 1.09)
Anticonvulsants, Yes vs. No		1.01 (0.67, 1.52)
ACEIs/ARBs, Yes vs. No		0.86 (0.54, 1.36)
β-blockers, Yes vs. No		0.70 (0.48, 1.01)
Tricyclic antidepressants, Yes vs. No		0.76 (0.50, 1.15)
Neurotoxins, Yes vs. No		0.51 (0.32, 0.80)
Number of prophylactic classes		1.28 (0.96, 1.72)
Hypertension, Yes vs. No		1.20 (0.91, 1.58)
Peripheral vascular disease, Yes vs. No		0.81 (0.43, 1.54)
Ischemic stroke/TIA, Yes vs. No		0.53 (0.27, 1.05)
Coronary artery disease, Yes vs. No		0.66 (0.41, 1.08)
Depression, Yes vs. No		1.09 (0.84, 1.41)

Abbreviations: ACEIs, angiotensin-converting enzyme inhibitors; ARBs, angiotensin II receptor blockers; TIA, transient ischemic attack.

© 2023 Wang K et al. *JAMA Network Open*.

eFigure. Distribution of Log-Transformed Stabilized Weight by Months

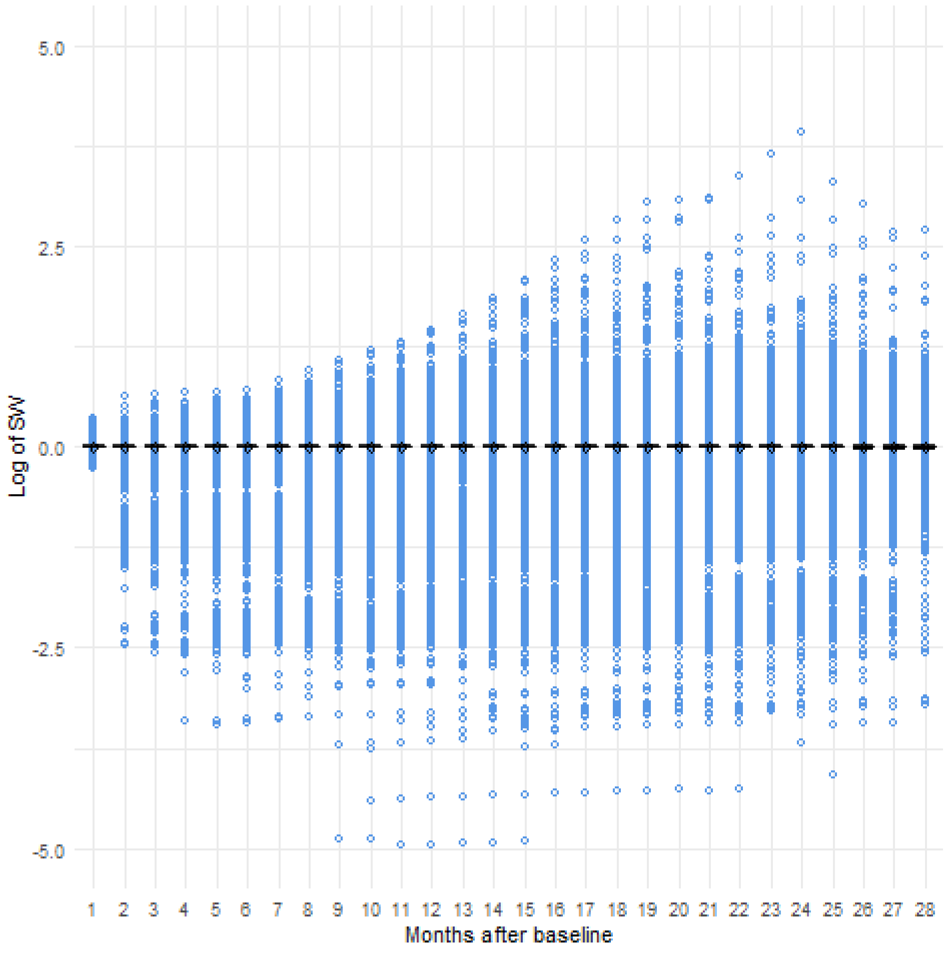

© 2023 Wang K et al. *JAMA Network Open*.
