# Supplementary material for: Calcitonin Gene–Related Peptide Monoclonal Antibodies and Risk of SARS-CoV-2 Infection and Severe COVID-19 Outcomes Among Veterans With Migraine Disorder
Source: JAMA Netw Open. 2023 Jul 31;6(7):e2326371. doi: 10.1001/jamanetworkopen.2023.26371 (PMC10391301; doi:10.1001/jamanetworkopen.2023.26371)
Supplement: Supplement 2. — Data Sharing Statement [file jamanetwopen-e2326371-s002.pdf]

Data Sharing Statement

Wang. Calcitonin Gene–Related Peptide Monoclonal Antibodies and Risk of SARS-CoV-2 Infection and Severe COVID-19 Outcomes Among Veterans With Migraine Disorder. *JAMA Netw Open*. Published July 31, 2023. doi:10.1001/jamanetworkopen.2023.26371

Data
Data available: Yes
Data types: Deidentified participant data
How to access data: The data used for this study are available with an approved study protocol by the Department of Veterans Affairs. The data are not publicly available due to regulations and ethics agreements. For more information, please visit https://www.virec.research.va.gov.
When available: With publication

Supporting Documents
Document types: None

Additional Information
Who can access the data: Investigators whose proposed use of the data has been approved by the Department of Veterans Affairs
Types of analyses: For any purpose of research
Mechanisms of data availability: After approval of a proposal and with a signed data access agreement
